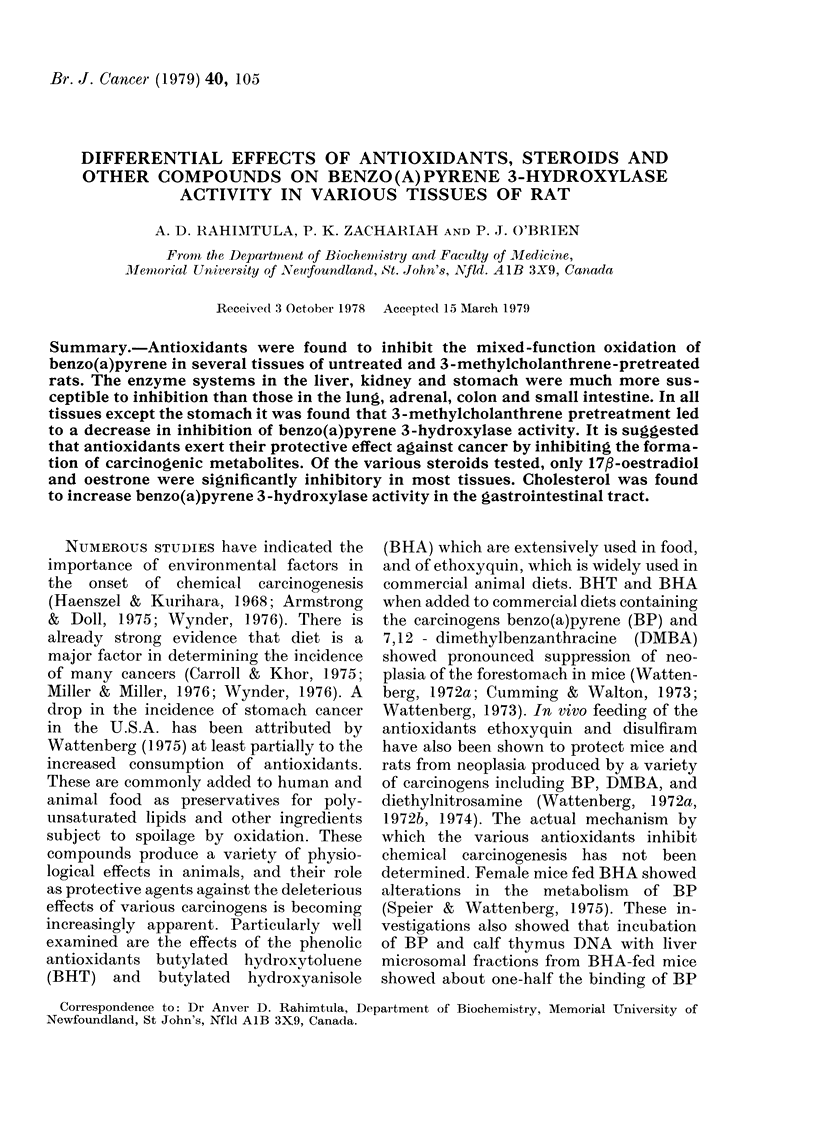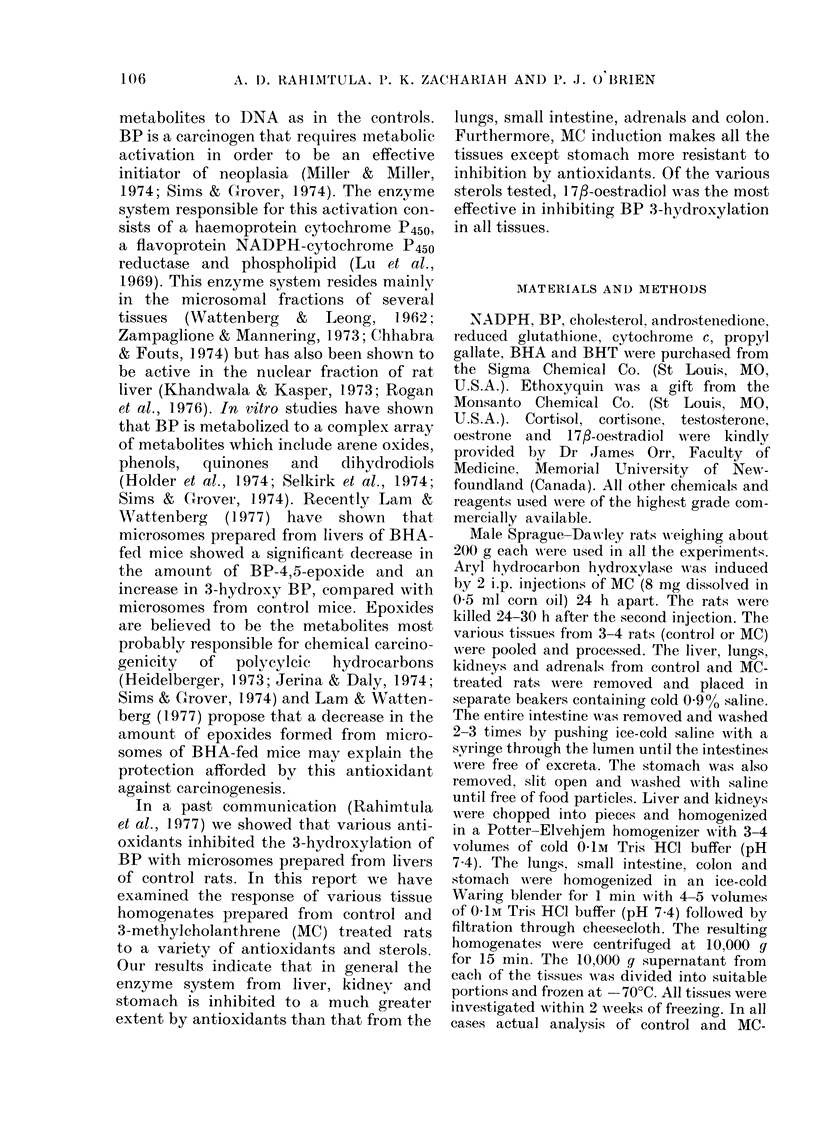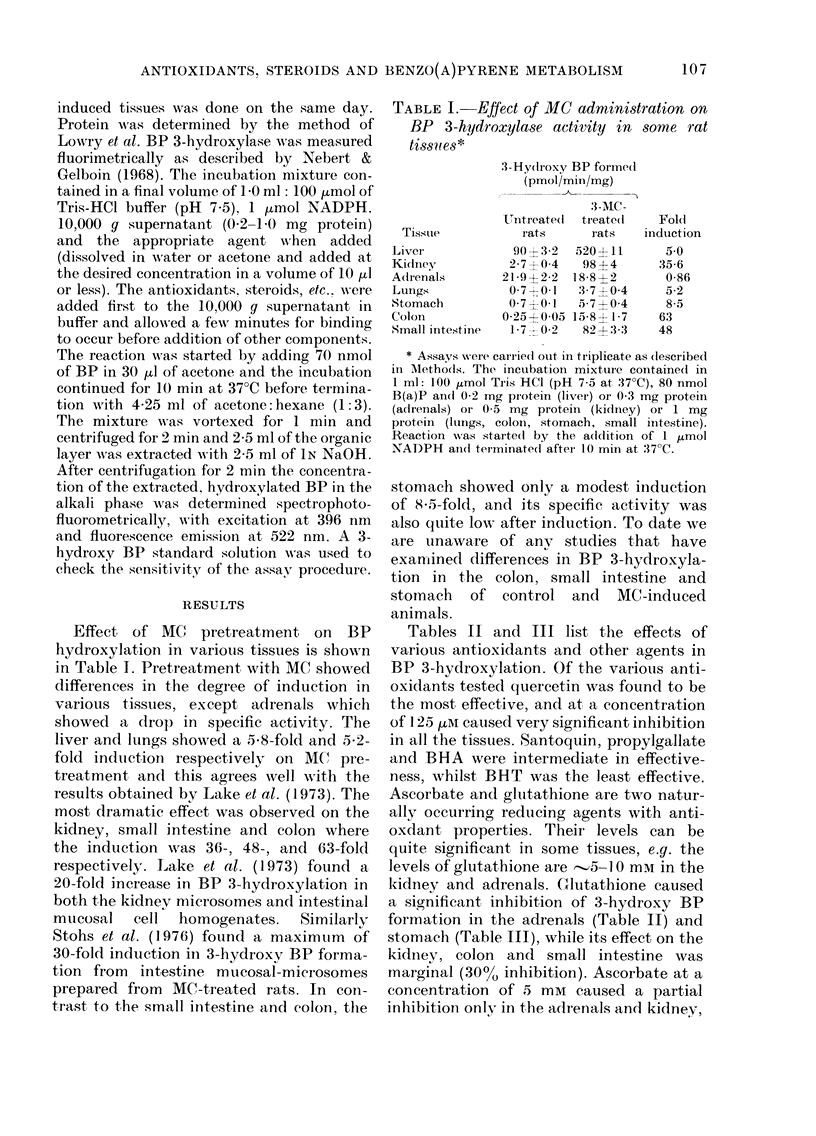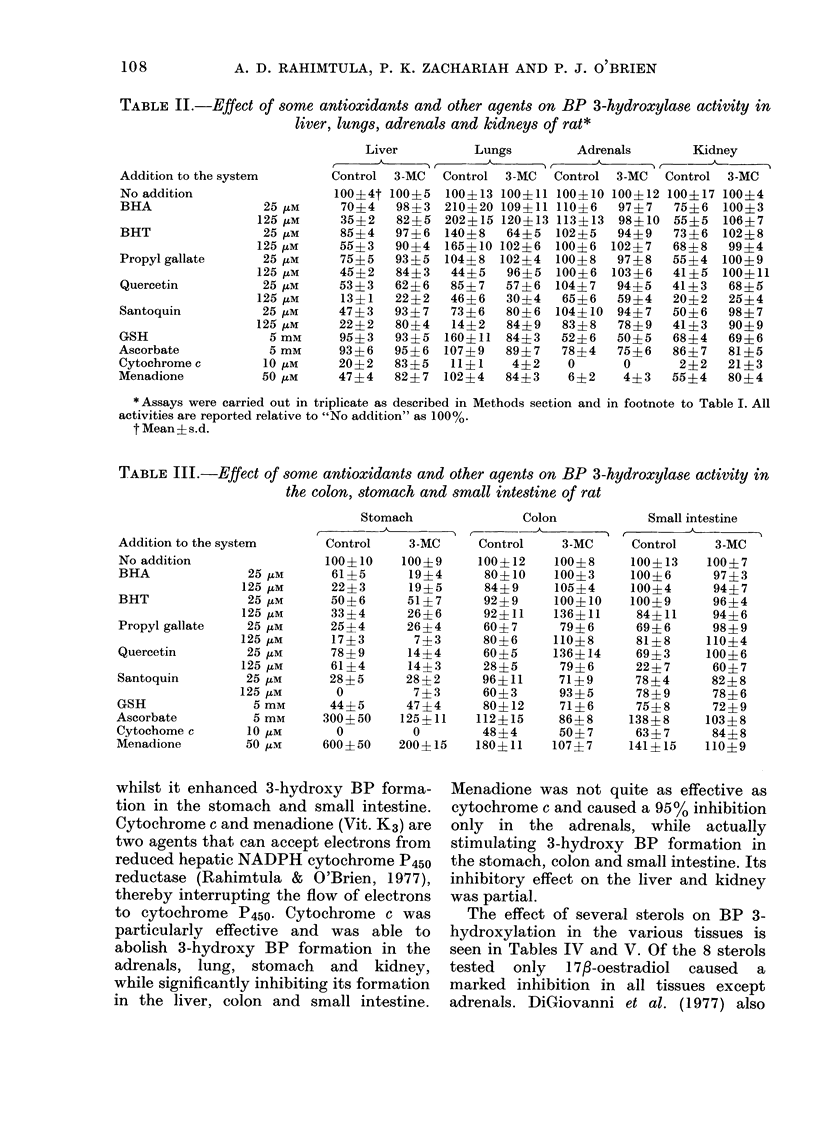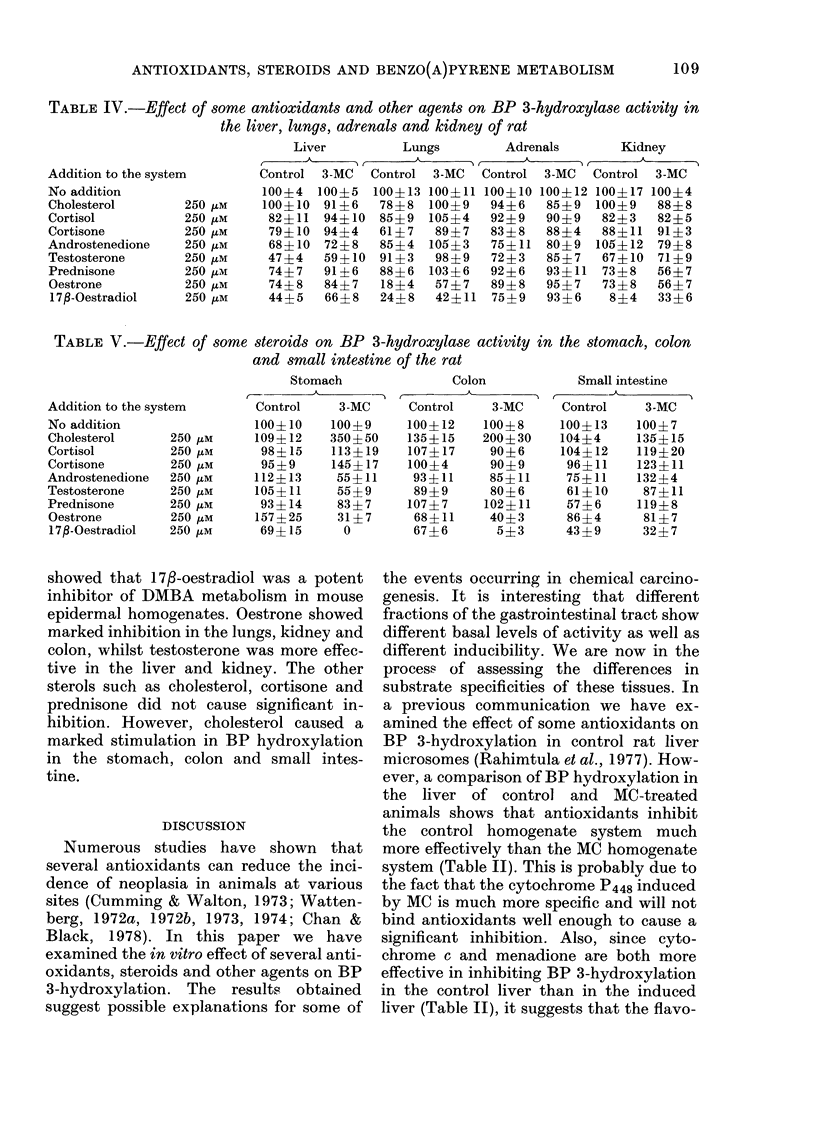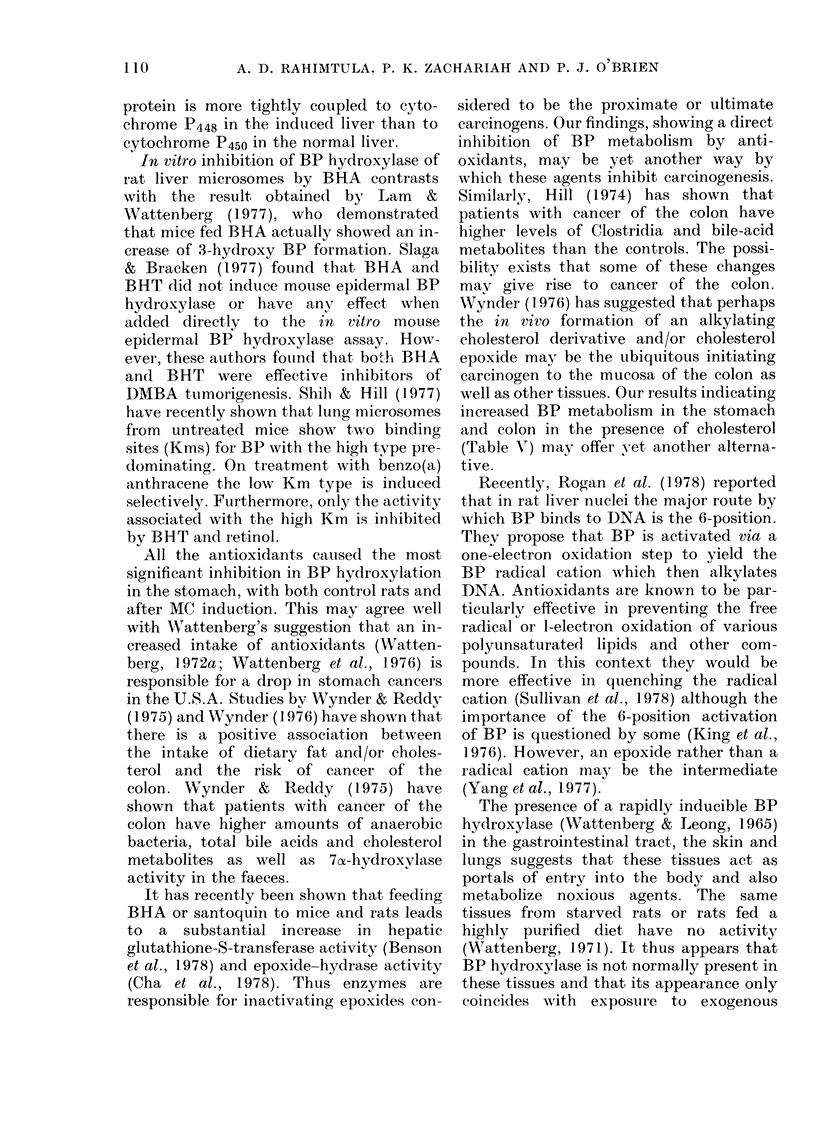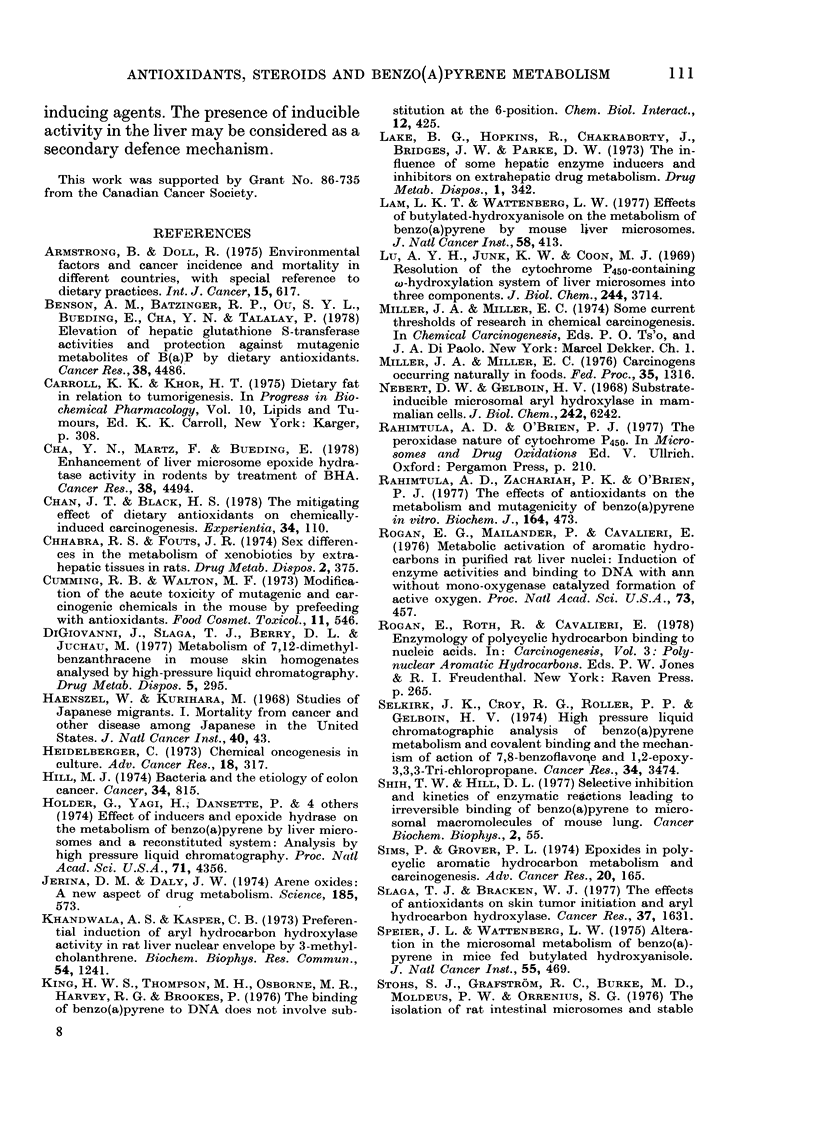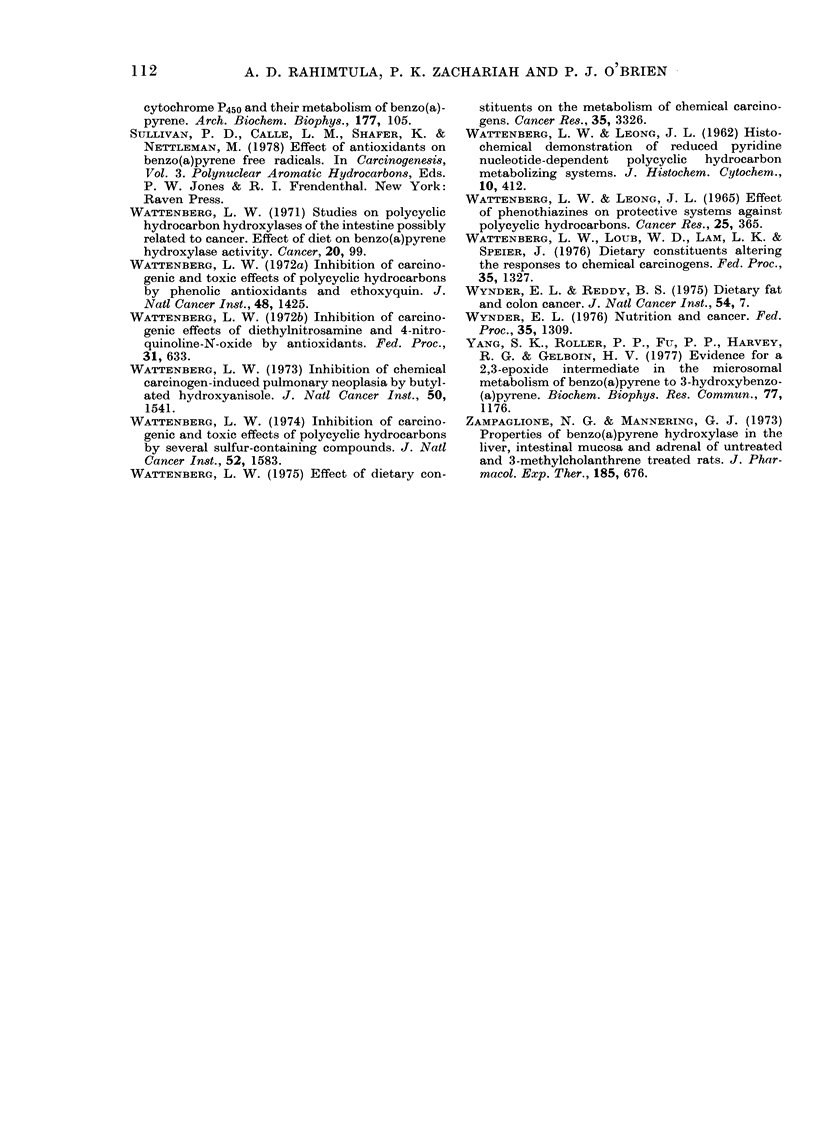# Differential effects of antioxidants, steroids and other compounds on benzo(a)pyrene 3-hydroxylase activity in various tissues of rat.

**DOI:** 10.1038/bjc.1979.146

**Published:** 1979-07

**Authors:** A. D. Rahimtula, P. K. Zachariah, P. J. O'Brien

## Abstract

Antioxidants were found to inhibit the mixed-function oxidation of benzo(a)pyrene in several tissues of untreated and 3-methylcholanthrene-pretreated rats. The enzyme systems in the liver, kidney and stomach were much more susceptible to inhibition than those in the lung, adrenal, colon and small intestine. In all tissues except the stomach it was found that 3-methylcholanthrene pretreatment led to a decrease in inhibition of benzo(a)pyrene 3-hydroxylase activity. It is suggested that antioxidants exert their protective effect against cancer by inhibiting the formation of carcinogenic metabolites. Of the various steroids tested, only 17 beta-oestradiol and oestrone were significantly inhibitory in most tissues. Cholesterol was found to increase benzo(a)pyrene 3-hydroxylase activity in the gastrointestinal tract.


*Br. J. Cancer* (1979) **40**, 105

### DIFFERENTIAL EFFECTS OF ANTIOXIDANTS, STEROIDS AND OTHER COMPOUNDS ON BENZO(A)PYRENE 3-HYDROXYLASE ACTIVITY IN VARIOUS TISSUES OF RAT

A. D. RAHIMTULA, P. K. ZACHARIAH AND P. J. O'BRIEN

*From the Department of Biochemistry and Faculty of Medicine, Memorial University of Newfoundland, St. John's, Nfld. A1B 3X9, Canada*

Received 3 October 1978 Accepted 15 March 1979

**Summary.—Antioxidants were found to inhibit the mixed-function oxidation of benzo(a)pyrene in several tissues of untreated and 3-methylcholanthrene-pretreated rats. The enzyme systems in the liver, kidney and stomach were much more susceptible to inhibition than those in the lung, adrenal, colon and small intestine. In all tissues except the stomach it was found that 3-methylcholanthrene pretreatment led to a decrease in inhibition of benzo(a)pyrene 3-hydroxylase activity. It is suggested that antioxidants exert their protective effect against cancer by inhibiting the formation of carcinogenic metabolites. Of the various steroids tested, only 17β-oestradiol and oestrone were significantly inhibitory in most tissues. Cholesterol was found to increase benzo(a)pyrene 3-hydroxylase activity in the gastrointestinal tract.**

NUMEROUS STUDIES have indicated the importance of environmental factors in the onset of chemical carcinogenesis (Haenszel & Kurihara, 1968; Armstrong & Doll, 1975; Wynder, 1976). There is already strong evidence that diet is a major factor in determining the incidence of many cancers (Carroll & Khor, 1975; Miller & Miller, 1976; Wynder, 1976). A drop in the incidence of stomach cancer in the U.S.A. has been attributed by Wattenberg (1975) at least partially to the increased consumption of antioxidants. These are commonly added to human and animal food as preservatives for polyunsaturated lipids and other ingredients subject to spoilage by oxidation. These compounds produce a variety of physiological effects in animals, and their role as protective agents against the deleterious effects of various carcinogens is becoming increasingly apparent. Particularly well examined are the effects of the phenolic antioxidants butylated hydroxytoluene (BHT) and butylated hydroxyanisole (BHA) which are extensively used in food, and of ethoxyquin, which is widely used in commercial animal diets. BHT and BHA when added to commercial diets containing the carcinogens benzo(a)pyrene (BP) and 7,12 - dimethylbenzanthracine (DMBA) showed pronounced suppression of neoplasia of the forestomach in mice (Wattenberg, 1972*a*; Cumming & Walton, 1973; Wattenberg, 1973). *In vivo* feeding of the antioxidants ethoxyquin and disulfiram have also been shown to protect mice and rats from neoplasia produced by a variety of carcinogens including BP, DMBA, and diethylnitrosamine (Wattenberg, 1972*a*, 1972*b*, 1974). The actual mechanism by which the various antioxidants inhibit chemical carcinogenesis has not been determined. Female mice fed BHA showed alterations in the metabolism of BP (Speier & Wattenberg, 1975). These investigations also showed that incubation of BP and calf thymus DNA with liver microsomal fractions from BHA-fed mice showed about one-half the binding of BP

Correspondence to: Dr Anver D. Rahimtula, Department of Biochemistry, Memorial University of Newfoundland, St John's, Nfld A1B 3X9, Canada.

metabolites to DNA as in the controls. BP is a carcinogen that requires metabolic activation in order to be an effective initiator of neoplasia (Miller & Miller, 1974; Sims & Grover, 1974). The enzyme system responsible for this activation consists of a haemoprotein cytochrome $P_{450}$, a flavoprotein NADPH-cytochrome $P_{450}$ reductase and phospholipid (Lu *et al.*, 1969). This enzyme system resides mainly in the microsomal fractions of several tissues (Wattenberg & Leong, 1962; Zampaglione & Mannering, 1973; Chhabra & Fouts, 1974) but has also been shown to be active in the nuclear fraction of rat liver (Khandwala & Kasper, 1973; Rogan *et al.*, 1976). *In vitro* studies have shown that BP is metabolized to a complex array of metabolites which include arene oxides, phenols, quinones and dihydrodiols (Holder *et al.*, 1974; Selkirk *et al.*, 1974; Sims & Grover, 1974). Recently Lam & Wattenberg (1977) have shown that microsomes prepared from livers of BHA-fed mice showed a significant decrease in the amount of BP-4,5-epoxide and an increase in 3-hydroxy BP, compared with microsomes from control mice. Epoxides are believed to be the metabolites most probably responsible for chemical carcinogenicity of polycylcic hydrocarbons (Heidelberger, 1973; Jerina & Daly, 1974; Sims & Grover, 1974) and Lam & Wattenberg (1977) propose that a decrease in the amount of epoxides formed from microsomes of BHA-fed mice may explain the protection afforded by this antioxidant against carcinogenesis.

In a past communication (Rahimtula *et al.*, 1977) we showed that various antioxidants inhibited the 3-hydroxylation of BP with microsomes prepared from livers of control rats. In this report we have examined the response of various tissue homogenates prepared from control and 3-methylcholanthrene (MC) treated rats to a variety of antioxidants and sterols. Our results indicate that in general the enzyme system from liver, kidney and stomach is inhibited to a much greater extent by antioxidants than that from the lungs, small intestine, adrenals and colon. Furthermore, MC induction makes all the tissues except stomach more resistant to inhibition by antioxidants. Of the various sterols tested, 17$\beta$-oestradiol was the most effective in inhibiting BP 3-hydroxylation in all tissues.

### MATERIALS AND METHODS

NADPH, BP, cholesterol, androstenedione, reduced glutathione, cytochrome *c*, propyl gallate, BHA and BHT were purchased from the Sigma Chemical Co. (St Louis, MO, U.S.A.). Ethoxyquin was a gift from the Monsanto Chemical Co. (St Louis, MO, U.S.A.). Cortisol, cortisone, testosterone, oestrone and 17$\beta$-oestradiol were kindly provided by Dr James Orr, Faculty of Medicine, Memorial University of Newfoundland (Canada). All other chemicals and reagents used were of the highest grade commercially available.

Male Sprague–Dawley rats weighing about 200 g each were used in all the experiments. Aryl hydrocarbon hydroxylase was induced by 2 i.p. injections of MC (8 mg dissolved in 0·5 ml corn oil) 24 h apart. The rats were killed 24–30 h after the second injection. The various tissues from 3–4 rats (control or MC) were pooled and processed. The liver, lungs, kidneys and adrenals from control and MC-treated rats were removed and placed in separate beakers containing cold 0·9% saline. The entire intestine was removed and washed 2–3 times by pushing ice-cold saline with a syringe through the lumen until the intestines were free of excreta. The stomach was also removed, slit open and washed with saline until free of food particles. Liver and kidneys were chopped into pieces and homogenized in a Potter–Elvehjem homogenizer with 3–4 volumes of cold 0·1M Tris HCl buffer (pH 7·4). The lungs, small intestine, colon and stomach were homogenized in an ice-cold Waring blender for 1 min with 4–5 volumes of 0·1M Tris HCl buffer (pH 7·4) followed by filtration through cheesecloth. The resulting homogenates were centrifuged at 10,000 *g* for 15 min. The 10,000 *g* supernatant from each of the tissues was divided into suitable portions and frozen at −70°C. All tissues were investigated within 2 weeks of freezing. In all cases actual analysis of control and MC-

induced tissues was done on the same day. Protein was determined by the method of Lowry *et al.* BP 3-hydroxylase was measured fluorimetrically as described by Nebert & Gelboin (1968). The incubation mixture contained in a final volume of 1·0 ml: 100 μmol of Tris-HCl buffer (pH 7·5), 1 μmol NADPH, 10,000 *g* supernatant (0·2–1·0 mg protein) and the appropriate agent when added (dissolved in water or acetone and added at the desired concentration in a volume of 10 μl or less). The antioxidants, steroids, *etc.*, were added first to the 10,000 *g* supernatant in buffer and allowed a few minutes for binding to occur before addition of other components. The reaction was started by adding 70 nmol of BP in 30 μl of acetone and the incubation continued for 10 min at 37°C before termination with 4·25 ml of acetone:hexane (1:3). The mixture was vortexed for 1 min and centrifuged for 2 min and 2·5 ml of the organic layer was extracted with 2·5 ml of 1N NaOH. After centrifugation for 2 min the concentration of the extracted, hydroxylated BP in the alkali phase was determined spectrophotofluorometrically, with excitation at 396 nm and fluorescence emission at 522 nm. A 3-hydroxy BP standard solution was used to check the sensitivity of the assay procedure.

### RESULTS

Effect of MC pretreatment on BP hydroxylation in various tissues is shown in Table I. Pretreatment with MC showed differences in the degree of induction in various tissues, except adrenals which showed a drop in specific activity. The liver and lungs showed a 5·8-fold and 5·2-fold induction respectively on MC pretreatment and this agrees well with the results obtained by Lake *et al.* (1973). The most dramatic effect was observed on the kidney, small intestine and colon where the induction was 36-, 48-, and 63-fold respectively. Lake *et al.* (1973) found a 20-fold increase in BP 3-hydroxylation in both the kidney microsomes and intestinal mucosal cell homogenates. Similarly Stohs *et al.* (1976) found a maximum of 30-fold induction in 3-hydroxy BP formation from intestine mucosal-microsomes prepared from MC-treated rats. In contrast to the small intestine and colon, the

TABLE I.—*Effect of MC administration on BP 3-hydroxylase activity in some rat tissues**

| Tissue | 3-Hydroxy BP formed (pmol/min/mg) Untreated rats | 3-MC-treated rats | Fold induction |
|---|---|---|---|
| Liver | 90±3·2 | 520±11 | 5·0 |
| Kidney | 2·7±0·4 | 98±4 | 35·6 |
| Adrenals | 21·9±2·2 | 18·8±2 | 0·86 |
| Lungs | 0·7±0·1 | 3·7±0·4 | 5·2 |
| Stomach | 0·7±0·1 | 5·7±0·4 | 8·5 |
| Colon | 0·25±0·05 | 15·8±1·7 | 63 |
| Small intestine | 1·7±0·2 | 82±3·3 | 48 |

* Assays were carried out in triplicate as described in Methods. The incubation mixture contained in 1 ml: 100 μmol Tris HCl (pH 7·5 at 37°C), 80 nmol B(a)P and 0·2 mg protein (liver) or 0·3 mg protein (adrenals) or 0·5 mg protein (kidney) or 1 mg protein (lungs, colon, stomach, small intestine). Reaction was started by the addition of 1 μmol NADPH and terminated after 10 min at 37°C.

stomach showed only a modest induction of 8·5-fold, and its specific activity was also quite low after induction. To date we are unaware of any studies that have examined differences in BP 3-hydroxylation in the colon, small intestine and stomach of control and MC-induced animals.

Tables II and III list the effects of various antioxidants and other agents in BP 3-hydroxylation. Of the various antioxidants tested quercetin was found to be the most effective, and at a concentration of 125 μM caused very significant inhibition in all the tissues. Santoquin, propylgallate and BHA were intermediate in effectiveness, whilst BHT was the least effective. Ascorbate and glutathione are two naturally occurring reducing agents with antioxdant properties. Their levels can be quite significant in some tissues, *e.g.* the levels of glutathione are ~5–10 mM in the kidney and adrenals. Glutathione caused a significant inhibition of 3-hydroxy BP formation in the adrenals (Table II) and stomach (Table III), while its effect on the kidney, colon and small intestine was marginal (30% inhibition). Ascorbate at a concentration of 5 mM caused a partial inhibition only in the adrenals and kidney,

TABLE II.—*Effect of some antioxidants and other agents on BP 3-hydroxylase activity in liver, lungs, adrenals and kidneys of rat**

| Addition to the system | | Liver Control | Liver 3-MC | Lungs Control | Lungs 3-MC | Adrenals Control | Adrenals 3-MC | Kidney Control | Kidney 3-MC |
|---|---|---|---|---|---|---|---|---|---|
| No addition | | 100±4† | 100±5 | 100±13 | 100±11 | 100±10 | 100±12 | 100±17 | 100±4 |
| BHA | 25 μM | 70±4 | 98±3 | 210±20 | 109±11 | 110±6 | 97±7 | 75±6 | 100±3 |
| | 125 μM | 35±2 | 82±5 | 202±15 | 120±13 | 113±13 | 98±10 | 55±5 | 106±7 |
| BHT | 25 μM | 85±4 | 97±6 | 140±8 | 64±5 | 102±5 | 94±9 | 73±6 | 102±8 |
| | 125 μM | 55±3 | 90±4 | 165±10 | 102±6 | 100±6 | 102±7 | 68±8 | 99±4 |
| Propyl gallate | 25 μM | 75±5 | 93±5 | 104±8 | 102±4 | 100±8 | 97±8 | 55±4 | 100±9 |
| | 125 μM | 45±2 | 84±3 | 44±5 | 96±5 | 100±6 | 103±6 | 41±5 | 100±11 |
| Quercetin | 25 μM | 53±3 | 62±6 | 85±7 | 57±6 | 104±7 | 94±5 | 41±3 | 68±5 |
| | 125 μM | 13±1 | 22±2 | 46±6 | 30±4 | 65±6 | 59±4 | 20±2 | 25±4 |
| Santoquin | 25 μM | 47±3 | 93±7 | 73±6 | 80±6 | 104±10 | 94±7 | 50±6 | 98±7 |
| | 125 μM | 22±2 | 80±4 | 14±2 | 84±9 | 83±8 | 78±9 | 41±3 | 90±9 |
| GSH | 5 mM | 95±3 | 93±5 | 160±11 | 84±3 | 52±6 | 50±5 | 68±4 | 69±6 |
| Ascorbate | 5 mM | 93±6 | 95±6 | 107±9 | 89±7 | 78±4 | 75±6 | 86±7 | 81±5 |
| Cytochrome *c* | 10 μM | 20±2 | 83±5 | 11±1 | 4±2 | 0 | 0 | 2±2 | 21±3 |
| Menadione | 50 μM | 47±4 | 82±7 | 102±4 | 84±3 | 6±2 | 4±3 | 55±4 | 80±4 |

* Assays were carried out in triplicate as described in Methods section and in footnote to Table I. All activities are reported relative to "No addition" as 100%.
† Mean±s.d.

TABLE III.—*Effect of some antioxidants and other agents on BP 3-hydroxylase activity in the colon, stomach and small intestine of rat*

| Addition to the system | | Stomach Control | Stomach 3-MC | Colon Control | Colon 3-MC | Small intestine Control | Small intestine 3-MC |
|---|---|---|---|---|---|---|---|
| No addition | | 100±10 | 100±9 | 100±12 | 100±8 | 100±13 | 100±7 |
| BHA | 25 μM | 61±5 | 19±4 | 80±10 | 100±3 | 100±6 | 97±3 |
| | 125 μM | 22±3 | 19±5 | 84±9 | 105±4 | 100±4 | 94±7 |
| BHT | 25 μM | 50±6 | 51±7 | 92±9 | 100±10 | 100±9 | 96±4 |
| | 125 μM | 33±4 | 26±6 | 92±11 | 136±11 | 84±11 | 94±6 |
| Propyl gallate | 25 μM | 25±4 | 26±4 | 60±7 | 79±6 | 69±6 | 98±9 |
| | 125 μM | 17±3 | 7±3 | 80±6 | 110±8 | 81±8 | 110±4 |
| Quercetin | 25 μM | 78±9 | 14±4 | 60±5 | 136±14 | 69±3 | 100±6 |
| | 125 μM | 61±4 | 14±3 | 28±5 | 79±6 | 22±7 | 60±7 |
| Santoquin | 25 μM | 28±5 | 28±2 | 96±11 | 71±9 | 78±4 | 82±8 |
| | 125 μM | 0 | 7±3 | 60±3 | 93±5 | 78±9 | 78±6 |
| GSH | 5 mM | 44±5 | 47±4 | 80±12 | 71±6 | 75±8 | 72±9 |
| Ascorbate | 5 mM | 300±50 | 125±11 | 112±15 | 86±8 | 138±8 | 103±8 |
| Cytochome *c* | 10 μM | 0 | 0 | 48±4 | 50±7 | 63±7 | 84±8 |
| Menadione | 50 μM | 600±50 | 200±15 | 180±11 | 107±7 | 141±15 | 110±9 |

whilst it enhanced 3-hydroxy BP formation in the stomach and small intestine. Cytochrome *c* and menadione (Vit. $K_3$) are two agents that can accept electrons from reduced hepatic NADPH cytochrome $P_{450}$ reductase (Rahimtula & O'Brien, 1977), thereby interrupting the flow of electrons to cytochrome $P_{450}$. Cytochrome *c* was particularly effective and was able to abolish 3-hydroxy BP formation in the adrenals, lung, stomach and kidney, while significantly inhibiting its formation in the liver, colon and small intestine. Menadione was not quite as effective as cytochrome *c* and caused a 95% inhibition only in the adrenals, while actually stimulating 3-hydroxy BP formation in the stomach, colon and small intestine. Its inhibitory effect on the liver and kidney was partial.

The effect of several sterols on BP 3-hydroxylation in the various tissues is seen in Tables IV and V. Of the 8 sterols tested only 17β-oestradiol caused a marked inhibition in all tissues except adrenals. DiGiovanni *et al.* (1977) also

TABLE IV.—*Effect of some antioxidants and other agents on BP 3-hydroxylase activity in the liver, lungs, adrenals and kidney of rat*

| | | Liver | | Lungs | | Adrenals | | Kidney | |
|---|---|---|---|---|---|---|---|---|---|
| Addition to the system | | Control | 3-MC | Control | 3-MC | Control | 3-MC | Control | 3-MC |
| No addition | | 100±4 | 100±5 | 100±13 | 100±11 | 100±10 | 100±12 | 100±17 | 100±4 |
| Cholesterol | 250 μM | 100±10 | 91±6 | 78±8 | 100±9 | 94±6 | 85±9 | 100±9 | 88±8 |
| Cortisol | 250 μM | 82±11 | 94±10 | 85±9 | 105±4 | 92±9 | 90±9 | 82±3 | 82±5 |
| Cortisone | 250 μM | 79±10 | 94±4 | 61±7 | 89±7 | 83±8 | 88±4 | 88±11 | 91±3 |
| Androstenedione | 250 μM | 68±10 | 72±8 | 85±4 | 105±3 | 75±11 | 80±9 | 105±12 | 79±8 |
| Testosterone | 250 μM | 47±4 | 59±10 | 91±3 | 98±9 | 72±3 | 85±7 | 67±10 | 71±9 |
| Prednisone | 250 μM | 74±7 | 91±6 | 88±6 | 103±6 | 92±6 | 93±11 | 73±8 | 56±7 |
| Oestrone | 250 μM | 74±8 | 84±7 | 18±4 | 57±7 | 89±8 | 95±7 | 73±8 | 56±7 |
| 17β-Oestradiol | 250 μM | 44±5 | 66±8 | 24±8 | 42±11 | 75±9 | 93±6 | 8±4 | 33±6 |

TABLE V.—*Effect of some steroids on BP 3-hydroxylase activity in the stomach, colon and small intestine of the rat*

| | | Stomach | | Colon | | Small intestine | |
|---|---|---|---|---|---|---|---|
| Addition to the system | | Control | 3-MC | Control | 3-MC | Control | 3-MC |
| No addition | | 100±10 | 100±9 | 100±12 | 100±8 | 100±13 | 100±7 |
| Cholesterol | 250 μM | 109±12 | 350±50 | 135±15 | 200±30 | 104±4 | 135±15 |
| Cortisol | 250 μM | 98±15 | 113±19 | 107±17 | 90±6 | 104±12 | 119±20 |
| Cortisone | 250 μM | 95±9 | 145±17 | 100±4 | 90±9 | 96±11 | 123±11 |
| Androstenedione | 250 μM | 112±13 | 55±11 | 93±11 | 85±11 | 75±11 | 132±4 |
| Testosterone | 250 μM | 105±11 | 55±9 | 89±9 | 80±6 | 61±10 | 87±11 |
| Prednisone | 250 μM | 93±14 | 83±7 | 107±7 | 102±11 | 57±6 | 119±8 |
| Oestrone | 250 μM | 157±25 | 31±7 | 68±11 | 40±3 | 86±4 | 81±7 |
| 17β-Oestradiol | 250 μM | 69±15 | 0 | 67±6 | 5±3 | 43±9 | 32±7 |

showed that 17β-oestradiol was a potent inhibitor of DMBA metabolism in mouse epidermal homogenates. Oestrone showed marked inhibition in the lungs, kidney and colon, whilst testosterone was more effective in the liver and kidney. The other sterols such as cholesterol, cortisone and prednisone did not cause significant inhibition. However, cholesterol caused a marked stimulation in BP hydroxylation in the stomach, colon and small intestine.

### DISCUSSION

Numerous studies have shown that several antioxidants can reduce the incidence of neoplasia in animals at various sites (Cumming & Walton, 1973; Wattenberg, 1972*a*, 1972*b*, 1973, 1974; Chan & Black, 1978). In this paper we have examined the *in vitro* effect of several antioxidants, steroids and other agents on BP 3-hydroxylation. The results obtained suggest possible explanations for some of the events occurring in chemical carcinogenesis. It is interesting that different fractions of the gastrointestinal tract show different basal levels of activity as well as different inducibility. We are now in the process of assessing the differences in substrate specificities of these tissues. In a previous communication we have examined the effect of some antioxidants on BP 3-hydroxylation in control rat liver microsomes (Rahimtula *et al.*, 1977). However, a comparison of BP hydroxylation in the liver of control and MC-treated animals shows that antioxidants inhibit the control homogenate system much more effectively than the MC homogenate system (Table II). This is probably due to the fact that the cytochrome $P_{448}$ induced by MC is much more specific and will not bind antioxidants well enough to cause a significant inhibition. Also, since cytochrome *c* and menadione are both more effective in inhibiting BP 3-hydroxylation in the control liver than in the induced liver (Table II), it suggests that the flavoprotein is more tightly coupled to cytochrome $P_{448}$ in the induced liver than to cytochrome $P_{450}$ in the normal liver.

*In vitro* inhibition of BP hydroxylase of rat liver microsomes by BHA contrasts with the result obtained by Lam & Wattenberg (1977), who demonstrated that mice fed BHA actually showed an increase of 3-hydroxy BP formation. Slaga & Bracken (1977) found that BHA and BHT did not induce mouse epidermal BP hydroxylase or have any effect when added directly to the *in vitro* mouse epidermal BP hydroxylase assay. However, these authors found that both BHA and BHT were effective inhibitors of DMBA tumorigenesis. Shih & Hill (1977) have recently shown that lung microsomes from untreated mice show two binding sites (Kms) for BP with the high type predominating. On treatment with benzo(a) anthracene the low Km type is induced selectively. Furthermore, only the activity associated with the high Km is inhibited by BHT and retinol.

All the antioxidants caused the most significant inhibition in BP hydroxylation in the stomach, with both control rats and after MC induction. This may agree well with Wattenberg's suggestion that an increased intake of antioxidants (Wattenberg, 1972*a*; Wattenberg *et al.*, 1976) is responsible for a drop in stomach cancers in the U.S.A. Studies by Wynder & Reddy (1975) and Wynder (1976) have shown that there is a positive association between the intake of dietary fat and/or cholesterol and the risk of cancer of the colon. Wynder & Reddy (1975) have shown that patients with cancer of the colon have higher amounts of anaerobic bacteria, total bile acids and cholesterol metabolites as well as 7α-hydroxylase activity in the faeces.

It has recently been shown that feeding BHA or santoquin to mice and rats leads to a substantial increase in hepatic glutathione-S-transferase activity (Benson *et al.*, 1978) and epoxide–hydrase activity (Cha *et al.*, 1978). Thus enzymes are responsible for inactivating epoxides considered to be the proximate or ultimate carcinogens. Our findings, showing a direct inhibition of BP metabolism by antioxidants, may be yet another way by which these agents inhibit carcinogenesis. Similarly, Hill (1974) has shown that patients with cancer of the colon have higher levels of Clostridia and bile-acid metabolites than the controls. The possibility exists that some of these changes may give rise to cancer of the colon. Wynder (1976) has suggested that perhaps the *in vivo* formation of an alkylating cholesterol derivative and/or cholesterol epoxide may be the ubiquitous initiating carcinogen to the mucosa of the colon as well as other tissues. Our results indicating increased BP metabolism in the stomach and colon in the presence of cholesterol (Table V) may offer yet another alternative.

Recently, Rogan *et al.* (1978) reported that in rat liver nuclei the major route by which BP binds to DNA is the 6-position. They propose that BP is activated *via* a one-electron oxidation step to yield the BP radical cation which then alkylates DNA. Antioxidants are known to be particularly effective in preventing the free radical or 1-electron oxidation of various polyunsaturated lipids and other compounds. In this context they would be more effective in quenching the radical cation (Sullivan *et al.*, 1978) although the importance of the 6-position activation of BP is questioned by some (King *et al.*, 1976). However, an epoxide rather than a radical cation may be the intermediate (Yang *et al.*, 1977).

The presence of a rapidly inducible BP hydroxylase (Wattenberg & Leong, 1965) in the gastrointestinal tract, the skin and lungs suggests that these tissues act as portals of entry into the body and also metabolize noxious agents. The same tissues from starved rats or rats fed a highly purified diet have no activity (Wattenberg, 1971). It thus appears that BP hydroxylase is not normally present in these tissues and that its appearance only coincides with exposure to exogenous inducing agents. The presence of inducible activity in the liver may be considered as a secondary defence mechanism.

This work was supported by Grant No. 86-735 from the Canadian Cancer Society.

### REFERENCES

ARMSTRONG, B. & DOLL, R. (1975) Environmental factors and cancer incidence and mortality in different countries, with special reference to dietary practices. *Int. J. Cancer*, **15**, 617.

BENSON, A. M., BATZINGER, R. P., OU, S. Y. L., BUEDING, E., CHA, Y. N. & TALALAY, P. (1978) Elevation of hepatic glutathione S-transferase activities and protection against mutagenic metabolites of B(a)P by dietary antioxidants. *Cancer Res.*, **38**, 4486.

CARROLL, K. K. & KHOR, H. T. (1975) Dietary fat in relation to tumorigenesis. In *Progress in Biochemical Pharmacology*, Vol. 10, Lipids and Tumours, Ed. K. K. Carroll, New York: Karger, p. 308.

CHA, Y. N., MARTZ, F. & BUEDING, E. (1978) Enhancement of liver microsome epoxide hydratase activity in rodents by treatment of BHA. *Cancer Res.*, **38**, 4494.

CHAN, J. T. & BLACK, H. S. (1978) The mitigating effect of dietary antioxidants on chemically-induced carcinogenesis. *Experientia*, **34**, 110.

CHHABRA, R. S. & FOUTS, J. R. (1974) Sex differences in the metabolism of xenobiotics by extrahepatic tissues in rats. *Drug Metab. Dispos.* **2**, 375.

CUMMING, R. B. & WALTON, M. F. (1973) Modification of the acute toxicity of mutagenic and carcinogenic chemicals in the mouse by prefeeding with antioxidants. *Food Cosmet. Toxicol.*, **11**, 546.

DIGIOVANNI, J., SLAGA, T. J., BERRY, D. L. & JUCHAU, M. (1977) Metabolism of 7,12-dimethylbenzanthracene in mouse skin homogenates analysed by high-pressure liquid chromatography. *Drug Metab. Dispos.* **5**, 295.

HAENSZEL, W. & KURIHARA, M. (1968) Studies of Japanese migrants. I. Mortality from cancer and other disease among Japanese in the United States. *J. Natl Cancer Inst.*, **40**, 43.

HEIDELBERGER, C. (1973) Chemical oncogenesis in culture. *Adv. Cancer Res.*, **18**, 317.

HILL, M. J. (1974) Bacteria and the etiology of colon cancer. *Cancer*, **34**, 815.

HOLDER, G., YAGI, H., DANSETTE, P. & 4 others (1974) Effect of inducers and epoxide hydrase on the metabolism of benzo(a)pyrene by liver microsomes and a reconstituted system: Analysis by high pressure liquid chromatography. *Proc. Natl Acad. Sci. U.S.A.*, **71**, 4356.

JERINA, D. M. & DALY, J. W. (1974) Arene oxides: A new aspect of drug metabolism. *Science*, **185**, 573.

KHANDWALA, A. S. & KASPER, C. B. (1973) Preferential induction of aryl hydrocarbon hydroxylase activity in rat liver nuclear envelope by 3-methylcholanthrene. *Biochem. Biophys. Res. Commun.*, **54**, 1241.

KING, H. W. S., THOMPSON, M. H., OSBORNE, M. R., HARVEY, R. G. & BROOKES, P. (1976) The binding of benzo(a)pyrene to DNA does not involve substitution at the 6-position. *Chem. Biol. Interact.*, **12**, 425.

LAKE, B. G., HOPKINS, R., CHAKRABORTY, J., BRIDGES, J. W. & PARKE, D. W. (1973) The influence of some hepatic enzyme inducers and inhibitors on extrahepatic drug metabolism. *Drug Metab. Dispos.*, **1**, 342.

LAM, L. K. T. & WATTENBERG, L. W. (1977) Effects of butylated-hydroxyanisole on the metabolism of benzo(a)pyrene by mouse liver microsomes. *J. Natl Cancer Inst.*, **58**, 413.

LU, A. Y. H., JUNK, K. W. & COON, M. J. (1969) Resolution of the cytochrome $P_{450}$-containing $\omega$-hydroxylation system of liver microsomes into three components. *J. Biol. Chem.*, **244**, 3714.

MILLER, J. A. & MILLER, E. C. (1974) Some current thresholds of research in chemical carcinogenesis. In *Chemical Carcinogenesis*, Eds. P. O. Ts'o, and J. A. Di Paolo. New York: Marcel Dekker. Ch. 1.

MILLER, J. A. & MILLER, E. C. (1976) Carcinogens occurring naturally in foods. *Fed. Proc.*, **35**, 1316.

NEBERT, D. W. & GELBOIN, H. V. (1968) Substrate-inducible microsomal aryl hydroxylase in mammalian cells. *J. Biol. Chem.*, **242**, 6242.

RAHIMTULA, A. D. & O'BRIEN, P. J. (1977) The peroxidase nature of cytochrome $P_{450}$. In *Microsomes and Drug Oxidations* Ed. V. Ullrich. Oxford: Pergamon Press, p. 210.

RAHIMTULA, A. D., ZACHARIAH, P. K. & O'BRIEN, P. J. (1977) The effects of antioxidants on the metabolism and mutagenicity of benzo(a)pyrene *in vitro*. *Biochem. J.*, **164**, 473.

ROGAN, E. G., MAILANDER, P. & CAVALIERI, E. (1976) Metabolic activation of aromatic hydrocarbons in purified rat liver nuclei: Induction of enzyme activities and binding to DNA with ann without mono-oxygenase catalyzed formation of active oxygen. *Proc. Natl Acad. Sci. U.S.A.*, **73**, 457.

ROGAN, E., ROTH, R. & CAVALIERI, E. (1978) Enzymology of polycyclic hydrocarbon binding to nucleic acids. In: *Carcinogenesis, Vol. 3: Polynuclear Aromatic Hydrocarbons*. Eds. P. W. Jones & R. I. Freudenthal. New York: Raven Press. p. 265.

SELKIRK, J. K., CROY, R. G., ROLLER, P. P. & GELBOIN, H. V. (1974) High pressure liquid chromatographic analysis of benzo(a)pyrene metabolism and covalent binding and the mechanism of action of 7,8-benzoflavone and 1,2-epoxy-3,3,3-Tri-chloropropane. *Cancer Res.*, **34**, 3474.

SHIH, T. W. & HILL, D. L. (1977) Selective inhibition and kinetics of enzymatic reactions leading to irreversible binding of benzo(a)pyrene to microsomal macromolecules of mouse lung. *Cancer Biochem. Biophys.*, **2**, 55.

SIMS, P. & GROVER, P. L. (1974) Epoxides in polycyclic aromatic hydrocarbon metabolism and carcinogenesis. *Adv. Cancer Res.*, **20**, 165.

SLAGA, T. J. & BRACKEN, W. J. (1977) The effects of antioxidants on skin tumor initiation and aryl hydrocarbon hydroxylase. *Cancer Res.*, **37**, 1631.

SPEIER, J. L. & WATTENBERG, L. W. (1975) Alteration in the microsomal metabolism of benzo(a)-pyrene in mice fed butylated hydroxyanisole. *J. Natl Cancer Inst.*, **55**, 469.

STOHS, S. J., GRAFSTRÖM, R. C., BURKE, M. D., MOLDEUS, P. W. & ORRENIUS, S. G. (1976) The isolation of rat intestinal microsomes and stable cytochrome $P_{450}$ and their metabolism of benzo(a)-pyrene. *Arch. Biochem. Biophys.*, **177**, 105.

SULLIVAN, P. D., CALLE, L. M., SHAFER, K. & NETTLEMAN, M. (1978) Effect of antioxidants on benzo(a)pyrene free radicals. In *Carcinogenesis, Vol.* 3. *Polynuclear Aromatic Hydrocarbons*, Eds. P. W. Jones & R. I. Frendenthal. New York: Raven Press.

WATTENBERG, L. W. (1971) Studies on polycyclic hydrocarbon hydroxylases of the intestine possibly related to cancer. Effect of diet on benzo(a)pyrene hydroxylase activity. *Cancer*, **20**, 99.

WATTENBERG, L. W. (1972*a*) Inhibition of carcinogenic and toxic effects of polycyclic hydrocarbons by phenolic antioxidants and ethoxyquin. *J. Natl Cancer Inst.*, **48**, 1425.

WATTENBERG, L. W. (1972*b*) Inhibition of carcinogenic effects of diethylnitrosamine and 4-nitroquinoline-N-oxide by antioxidants. *Fed. Proc.*, **31**, 633.

WATTENBERG, L. W. (1973) Inhibition of chemical carcinogen-induced pulmonary neoplasia by butylated hydroxyanisole. *J. Natl Cancer Inst.*, **50**, 1541.

WATTENBERG, L. W. (1974) Inhibition of carcinogenic and toxic effects of polycyclic hydrocarbons by several sulfur-containing compounds. *J. Natl Cancer Inst.*, **52**, 1583.

WATTENBERG, L. W. (1975) Effect of dietary constituents on the metabolism of chemical carcinogens. *Cancer Res.*, **35**, 3326.

WATTENBERG, L. W. & LEONG, J. L. (1962) Histochemical demonstration of reduced pyridine nucleotide-dependent polycyclic hydrocarbon metabolizing systems. *J. Histochem. Cytochem.*, **10**, 412.

WATTENBERG, L. W. & LEONG, J. L. (1965) Effect of phenothiazines on protective systems against polycyclic hydrocarbons. *Cancer Res.*, **25**, 365.

WATTENBERG, L. W., LOUB, W. D., LAM, L. K. & SPEIER, J. (1976) Dietary constituents altering the responses to chemical carcinogens. *Fed. Proc.*, **35**, 1327.

WYNDER, E. L. & REDDY, B. S. (1975) Dietary fat and colon cancer. *J. Natl Cancer Inst.*, **54**, 7.

WYNDER, E. L. (1976) Nutrition and cancer. *Fed. Proc.*, **35**, 1309.

YANG, S. K., ROLLER, P. P., FU, P. P., HARVEY, R. G. & GELBOIN, H. V. (1977) Evidence for a 2,3-epoxide intermediate in the microsomal metabolism of benzo(a)pyrene to 3-hydroxybenzo-(a)pyrene. *Biochem. Biophys. Res. Commun.*, **77**, 1176.

ZAMPAGLIONE, N. G. & MANNERING, G. J. (1973) Properties of benzo(a)pyrene hydroxylase in the liver, intestinal mucosa and adrenal of untreated and 3-methylcholanthrene treated rats. *J. Pharmacol. Exp. Ther.*, **185**, 676.